# Anticancer Drug Approval in China: From Acceleration of Access to Certainty of Benefits

**DOI:** 10.34133/hds.0339

**Published:** 2025-10-28

**Authors:** Jianrong Zhang

**Affiliations:** ^1^ Victorian Cancer Registry, Cancer Council Victoria, Melbourne, Australia.; ^2^ School of Health & Wellbeing, College of Medical, Veterinary & Life Sciences, University of Glasgow, Glasgow, UK.

I read with great interest about a study that assessed the characteristics of pivotal trials with innovative and modified new anticancer drugs approved in China [1]. In this letter, I reflect on the study designs of these trials, given the context of oncology trial development in China and internationally.

As found in this study, the trials for innovative drugs are more likely to be single-arm, nonrandomized, open-label, and without an active comparator when compared to the trials for modified new drugs [1]. Although the rationale is allowing faster trial completion and approval of these drugs to treat unmet medical needs, a contradictory fact of concern is that these trials had no differences in the time for trial conduct, the number of patients in the intervention group, and treatment effects and had a longer time for approval review, as also found in this study [1].

Given these results, a question to be asked is whether there is still a strong need for the compromise aimed at accelerating trial completion and approval despite the lower scientific rigor. The evidence against the compromise also includes the rapid growth and current scale of Chinese drug development capacity, in terms of the number and timeliness of new cancer drugs launched [2], as well as the increasing participation in the global development of innovative anticancer drugs [3], suggesting that the unmet needs might no longer be urgent. Other evidence includes international experiences that (a) a large number of accelerated approvals for anticancer drugs and indications have been withdrawn recently [4] and that (b) most of these anticancer drugs were found to have no benefits in overall survival (OS) or quality of life within 5 years of accelerated approval [5]. Echoing the second point, no OS benefit was also found among the trials for modified new drugs in the pooled analysis of this study, despite these trials being more likely to be scientifically rigorous [1]. Notably, this result is based on only 5 of the 37 trials with a comparator; the availability of OS (and quality of life) may be another issue, which has been observed in international trials receiving accelerated approval and using surrogate endpoints [4,5].

Internationally, aiming for a higher certainty of clinical benefits from clinical trials is the right direction. Along with requiring more rigorous designs, future efforts in China may include implementing robust postmarketing surveillance and further improving the availability and affordability of anticancer drugs through healthcare coverage, price negotiation, and investments in the domestic industry.
